# 3D patient-derived tumor models to recapitulate pediatric brain tumors *In Vitro*

**DOI:** 10.1016/j.tranon.2022.101407

**Published:** 2022-04-02

**Authors:** Min D. Tang-Schomer, Harshpreet Chandok, Wei-Biao Wu, Ching C. Lau, Markus J. Bookland, Joshy George

**Affiliations:** a Connecticut Children's Medical Center, 282 Washington St, Hartford, CT 06106, USA; b UConn Health, Department of Pediatrics, 263 Farmington Avenue, Farmington, Connecticut 06030, USA; c The Jackson Laboratory for Genomic Medicine, 10 Discovery Drive, Farmington, Connecticut 06030, USA; d University of Chicago, Department of Statistics, 5747 S.Ellis Avenue, Chicago, IL 60637, USA

**Keywords:** Pediatric brain tumor, Brain model, Tumor microenvironment, Personalized medicine, Transcriptomic profiles

## Abstract

Brain tumors are the leading cause of cancer-related deaths in children. Tailored therapies need preclinical brain tumor models representing a wide range of molecular subtypes. Here, we adapted a previously established brain tissue-model to fresh patient tumor cells with the goal of establishing3D *in vitro* culture conditions for each tumor type.Wereported our findings from 11 pediatric tumor cases, consisting of three medulloblastoma (MB) patients, three ependymoma (EPN) patients, one glioblastoma (GBM) patient, and four juvenile pilocytic astrocytoma (Ast) patients. Chemically defined media consisting of a mixture of pro-neural and pro-endothelial cell culture medium was found to support better growth than serum-containing medium for all the tumor cases we tested. 3D scaffold alone was found to support cell heterogeneity and tumor type-dependent spheroid-forming ability; both properties were lost in 2D or gel-only control cultures. Limited *in vitro* models showed that the number of differentially expressed genes between *in vitro vs.* primary tissues, are 104 (0.6%) of medulloblastoma, 3,392 (20.2%) of ependymoma, and 576 (3.4%) of astrocytoma, out of total 16,795 protein-coding genes and lincRNAs. Two models derived from a same medulloblastoma patient clustered together with the patient-matched primary tumor tissue; both models were 3D scaffold-only in Neurobasal and EGM 1:1 (v/v) mixture and differed by a 1-mo gap in culture (i.e., 6wk versus 10wk). The genes underlying the *in vitro*vs. *in vivo* tissue differences may provide mechanistic insights into the tumor microenvironment. This study is the first step towards establishing a pipeline from patient cells to models to personalized drug testing for brain cancer.

## Highlights

• In vitro 3D medulloblastoma models have indistinguishable transcriptomic profiles than the original tumor tissue.
• One type of chemically defined media was identified to support four major types of pediatric brain tumor better than serum-containing culture conditions.
• Different tumor types show varying spheroid-forming ability depending on stem cell composition in the original tumor cell population.
• Different tumor types show different preference to 3D scaffold and/or extracellular matrix for the *in vitro* growth accompanied by microenvironment-induced gene expression changes.

## Introduction

Brain tumors are the leading cause of cancer-related deaths in children, and more than 4,600 children will be diagnosed with a brain tumor this year in the United States. Despite significant advances in the treatment of pediatric malignancies, the overall survival rate for certain brain tumors, such as medulloblastoma and glioblastoma, remains poor [1,2] . Since current therapeutic approaches fail in more than 20% of patients and the survivors acquire long-term cognitive and physical disabilities from treatment, new and more specific therapies are urgently needed. However, specific genetic makeups of different pediatric brain tumor types are incomplete and actionable targets are missing. Genome-wide profiling efforts continue to define subgroups with distinct prognoses both within and across pediatric brain tumor entities. Pediatric brain tumors include embryonal tumors such as medulloblastoma (MB); high-grade glioma (HGG), including glioblastoma (GBM); ependymoma (EPN); and astrocytoma (Ast). The last group encompasses many subtypes including some ‘low-grade’ types (e.g., LGG) that can transform into more malignant gliomas [3]. Medulloblastoma, one of the most frequent malignant brain tumors in children, consists of at least four distinct molecular subgroups: WNT, sonic hedgehog (SHH), Group 3, and Group 4 [4]. The molecular pathways driving Group 3 and Group 4 subtypes are unknown and have no targeted therapy, resulting in poor patient outcomes. Ependymoma, the third most common brain tumor in children and incurable in more than half of the cases, presents considerable histopathological variation and biological heterogeneity [5]. These challenges underscore the need for reliable preclinical model systems that retain the molecular characteristics of pediatric brain tumors for scientific research.

*In vitro* tumor culture models are indispensable preclinical tools for drug screening and therapeutic development. Yet, compared to the commonly used adult cell lines, there is a dearth of cell lines derived from children [11,12]. Because pediatric brain tumors respond differently to treatment compared to adults [13,14], there is a sore need for cell sources representative of childhood brain tumor subtypes [8]. However, most commercially available brain tumor cell lines have been propagated for decades in cell culture [15]. Clonal selection and genetic drift occur during serial passage of tumor cells as they adapt to monolayer cultures. Consequently, the original molecular features and biological behavior of the tumor cells have been lost [16]. These observations suggest that established cell lines are poor models for pediatric brain tumors.

With primary brain tumor cultures, it is increasingly clear that plastic dish-based 2D cultures do not support all tumor types especially of the lower grade tumor, nor preserve the primary cells’ properties faithfully after multiple passages. To address this problem, 3D neurospheres or spheroids have been developed based on the self-assembly of tumor cell aggregates [17] or clonal expansion of cancer stem cells [18], [19], [20]. These 3D tumor models are found to recapitulate 3D cell-cell and cell-matrix interactions and transport properties [19], [20], [21], thus promoting*in vivo*-like tumor behavior. For example, glioma spheroids more closely recapitulate the molecular makeup of the parental tumor and present more stable molecular profiles over time compared to 2D cultures [22,23]. 3D tumor spheroids can develop a hypoxic inner core region mimicking *in vivo* solid tumors that contribute to more realistic drug sensitivity compared to 2D monolayers [24], [25], [26]. However, the sphere-forming ability is limited to certain tumor types, inconsistent among individual tumor cases, and thought to depend on the proportion of cancer stem cells contained in the total cell population [10]. Patient-derived xenograft mouse models have been developed using patient tumor biopsies [[6], [7], [8], [9]]. However, the mouse brain microenvironment precludes the understanding of the human brain microenvironment that provides critical support for the malignant transformation and progression of human brain tumors.

In this study, we attempted to reconstitute a brain tumor from a patient's primary tumor tissue in 3D *in vitro* models to match the parental tumor's molecular characteristics as much as possible. The brain tumor models are based on a normal brain cortical tissue model we have established using a 3D silk fibroin-based porous scaffold as the structural base [27], [28], [29], [30], [31]. In our previous studies, we have optimized methods for ECM incorporation, cell seeding, and prolonged (months) culture for long-term model growth and *in vivo*-like brain tissue responses to physiological stimuli. We extended the silk scaffold-based model system to support patients’ brain cells isolated from epileptic surgeries at Connecticut Children's Medical Center (CCMC) and generated 3D human brain cell cultures with regeneration-like growth [32]. While inert, the versatile silk scaffold structure can be tailored to incorporate different ECM components [33] and multiple cell types [34,35] to better mimic *in vivo*brain tissue's complexity. Our earlier work of recapitulating human patients’ brain tumors using this system has illuminated a primary tumor's response to different ECM microenvironments [35]. This study aims to define the starting condition of 3D models (e.g., ECM, culture media, cell initial seeding) for different brain tumor types as encountered by the pediatric population. The *in vitro*models were evaluated by comparing their genome-wide expression profiles with matched parental tumor. These findings will guide future bioengineering approaches to develop patient-derived 3D brain tumor models for personalized disease and drug testing studies.

## Materials and methods

### Patient brain tumor tissue

Human patient brain tissue was obtained from tumor resection neurosurgery in Connecticut Children's Medical Center (CCMC) at Hartford, Connecticut. The procedures were approved by the Institutional Review Boards of UConn Health Center and CCMC (IRB #13-035). Informed consent was obtained from all human patients prior to the surgery. All methods were performed in accordance with the guidelines and regulations by the approved IRB protocol. Tissue specimen was transported in chilled RPMI-1640 medium (Sigma-Aldrich, St Louis, MO, USA) containing 1% penicillin-streptomycin (Pen/Strep, Thermo Fisher, Waltham, MA, USA) and 5% fetal bovine serum (FBS) on an ice pack from the operation room to the laboratory in <4-hour post-surgery.

### Brain tumor tissue dissociation

The tissue specimen was weighed, cut into ∼1 mm^3^ pieces with a sterile razor blade, re-suspended at 1,600 mg tissue/10 mL in Hibernate-A medium ^55^ (Thermo Fisher) containing 1% Pen/Strep and primocin (10 µg/mL, InvivoGen, San Diego, CA, USA). The tissue suspension was treated with a cocktail of enzymes (DNase I, 50 U; dispase II, 5 U; collagenase I, 1 U and collagenase IV, 10 mg/mL in 10 mL 0.5% Trypsin-EDTA solution) at 37°C for 20 min, followed by neutralization with a 10 mL trypsin inhibitor (0.5%, w/v) solution and gentle pipetting. The tissue dissociation solution was filtered with 100-µm cell strainer (Fisher Scientific, Suwannee, GA, USA) and single cell suspension was collected.

### 3D Silk protein-based scaffolds and extracellular matrix (ECM) gel infusion

Silk solution and porous scaffolds were prepared from *Bombyxmori*cocoons as described previously [[27], [31]]. A biopsy punch was used to generate donut-shaped silk protein-based scaffold (outer dia. 5 mm; inner dia. 2 mm; height, 2 mm). Silk scaffolds were autoclaved, coated with poly-D-lysine (10 µg/mL, Sigma) overnight, and washed 3 times with phosphate buffered saline (PBS, Sigma). Collagen gel was prepared from rat tail type I collagen (3-4 mg/ml, Fisher Scientific), 10X M199 medium (Thermo Fisher) and 1 M sodium hydroxide mixed at a ratio of 88:10:2, followed by gelling at 37°C for 1 hr. Matrigel (∼ 10 mg/mL, growth factor reduced, Fisher Scientific) was mixed at 1:1 ratio with collagen gel solution (8-10 mg/ml) before infusing the silk scaffolds. To make a scaffold-gel composite structure, the cell-seeded scaffolds were infused with the liquid ECM gel and incubated at 37°C for 1 hour for the gel to solidify before culture medium immersion.

### Cell plating

For 2D cultures, cells were plated at 250,000 cells/well in 6-well plates. For 3D scaffold-based cultures, the scaffolds were immersed in high-density cell suspensions (∼ 100 million cells/mL) for 24 hours followed by extensive washes with media and proceed to scaffold-only cultures or ECM gel-infused composite cultures. Culture media used include: NeuralBasal\B27 (Invitrogen, Grand Island, NY, USA) supplemented with 20 ng/mL recombinant human fibroblast growth factor, basic-154 (FGF, ConnStem, Cheshire, CT, USA) and 20 ng/mL human epidermal growth factor (EGF, PeproTech, Rocky Hill, NJ, USA), termed “NB” medium; NeuralBasal\B27\EGF\FGF supplemented with 10% fetal bovine serum (FBS, Denville Scientific, Metuchen, NJ, USA) (“N+FBS”), and “NB” medium mixed at 1:1 with endothelial growth media EGM-2MV (Lonza, Walkersville, MD, USA) without serum (“NE”). Media were changed once a week for all culture systems.

### Tissue viability assay

AlamarBlue assay was used to measure cell viability of 3D cultures, according to the manufacturer's protocol (Thermo Fisher Scientific). Briefly, alamarBlue reagent was mixed in fresh culture media (1:10, v/v) and incubated for 2 hrs at 37°C. The solution was transferred into a new 96-well plate. The fluorescence intensity was read at Ex./Em. of 560/590 nm on a micro-plate spectrophotometer (Synergy 2, BioTek, VT, USA). Four replicate cultures per condition were used for this assay, and the readings were normalized against media controls.

### Live/Dead assay

Live/Dead Assay was used to image live cells in cultures, according to the manufacturer's protocol (Thermo Fisher Scientific). Briefly, cultures were washed once with PBS, incubated with fresh medium containing calcein-AM (Live stain, 2 µg/mL) and ethidium homodimer-1 (Dead stain, 1 µg/mL) at 37°C for 20 min, washed with PBS once, and returned to fresh medium for another 20 min at 37°C. The stained cultures were imaged with a fluorescence microscope at Ex/Em of 494/517 nm and 528/617 nm for Live and Dead stains, respectively.

### Immunofluorescence staining and imaging

Cell cultures were fixed with 4% paraformaldehyde (Electron Microscopy Sciences, Hatfield, PA, USA) for 20 min, washed, permeabilized with PBS containing 0.1% Triton X-100 (Fisher Scientific) and 4% normal goat serum (Jackson ImmunoResearch Labs, West Grove, PA, USA) for 20 min, followed with incubation of primary antibodies overnight at 4°C. After three 10 min PBS washes, cells were incubated with secondary antibodies for 1 hr at room temperature, followed by three washes. Antibodies included: anti-βIII-tubulin (TUJ1, mouse clone 2G10, 1:500, eBioscience; rabbit, Sigma), anti-glial fibrillary acidic protein (GFAP, mouse clone GA5, 1:500, eBioscience; rabbit, Thermo Fisher), anti-Nestin (mouse clone 10C2, 1:100, eBioscience), anti-Ki67 (mouse clone B56, 1:100, BD Biosciences), anti-Vimentin (mouse clone RV202, 1:200, BD Bioscience). Goat anti-mouse or rabbit Alexa 488 and 568 (1:250; Invitrogen) secondary antibodies were used. Fluorescence images were acquired on a Leica DM IL fluorescence microscope using excitation/emission (Ex/Em) of 470/525 nm for Alexa 488, and Ex/Em of 560/645 nm for Alexa 568. Confocal images were acquired on a Zeiss 780 laser scanning confocal imaging system.

### Flow cytometry

Cells were treated with 0.5% trypsin-EDTA (Invitrogen) for 5 min or 25 min for 2D and 3D cultures, respectively. Cell suspensions were mixed at 1:1 with medium containing 10% FBS, and centrifuged at 300 g, 5 min. Cell pellets were re-suspended in PBS containing 2% FBS and stained on ice for 15 min with eFluor 780 (AffymetrixeBioscience, San Diego, CA, USA). Cells were washed in 2% FBS-containing PBS by centrifuging at 300 g, 5 min. Cell pellets were re-suspended, and stained with membrane-bound flow antibodies on ice for 20 min, and washed. Stained/washed cells were fixed with 4% paraformaldehyde for 20 min, washed, and permeabilized with PBS containing 0.1% Tween and 2% FBS for 20 min. Cells were subsequently stained with intracellular flow antibodies for 30 min, washed, and proceeded to flow cytometry. Flow antibodies used include: anti-human Ki67-eFluor 450, Nestin-Alexa 594, TUJ1-Alexa 488, GFAP-Alexa 647, Vimentin or CD133-PE, CD56-BUV 395 (all antibodies were from BioLegend, San Diego, CA, USA). Flow cytometry was performed on a BD LSR II instrument equipped with 5-lasers with BD FACS DIVA software (BD Biosciences). 2,000 - 5,000 cells per sample were counted and analyzed with FlowJo software (FlowJo, Ashland, OR, USA). Un-stained cells were used to set a gate for “single” cells. eFluor 780-stained cells were used to set the gate for “live” cells and “control” gates for each stain with a threshold of 0.5% (e.g., <0.5% cells were positive for the respective stain). Positive cell population corresponding to a stain was calculated from the multiplexed cell population using the same gate as that used for the control un-stained cell population.

### RNA-seq and transcriptomic profiling

RNAs were extracted with QiageneAllPrep kit on a QiaCube automated station. Samples were sequenced by JAX-GM Genome Technologies Core. RNA-seq libraries were prepared with KAPA Stranded mRNA-Seq kit. Quantification of libraries was performed using real-time qPCR. Sequencing was performed on IlluminaHiseq 4000 platform generating paired-end reads of 75bp. Raw reads obtained from the sequencer were processed including quality control steps to identify and remove low-quality samples. Reads with more than 50% low-quality bases (>Q30) overall were filtered out and the remaining high-quality reads were then used for expression estimation. Alignment estimation of gene expression levels using the EM algorithm for paired-end read data was performed using RSEM (package version 1.2.19) [36], with default settings. Bowtie2 was used as an aligner to align the mapped reads against the hg38 reference genome. Data quality control was performed using picardand bamtools to obtain general alignment statistics from the bam file. The expected counts data were normalized using the TMM normalization method. Differential expression gene (DEG) analysis to compare the expression profiles between *in vitro* cultures and the primary tissues was conducted using edgeR package in R [37]. For principle component analysis (PCA), the list of genes was selected based on variance, of which the lower 10% of variables were removed using PCAtools. Principal components (PC) were represented using bi-plot. For the heatmaps, the top 50 ranked genes based on each axis (e.g, PC-1 and PC-2) were used for the plots. GSEA (Gene Set Enrichment Analysis) was performed using the entire filtered gene list n=16,795. Log transformed normalized counts (cpm- counts per million) were used to obtain enriched KEGG pathways or enriched pathways using “hallmark” gene sets as part of MSigDB, with pvalue< 0.05.

### Statistical analysis

Data are mean ± standard error of the mean (S.E.M.), except where otherwise noted. Analysis used Student's t-test, except cell percentage data. For all tests, *p*< 0.05 was considered significant. For statistical analysis of flow cytometry-measured cell percentages, construction of simultaneous confidence intervals was performed, as we previously described [32]. A program written in R was used to implement the analysis

## Results

### Study design of the optimization of 3D cell culture conditions

**Figure 1:**
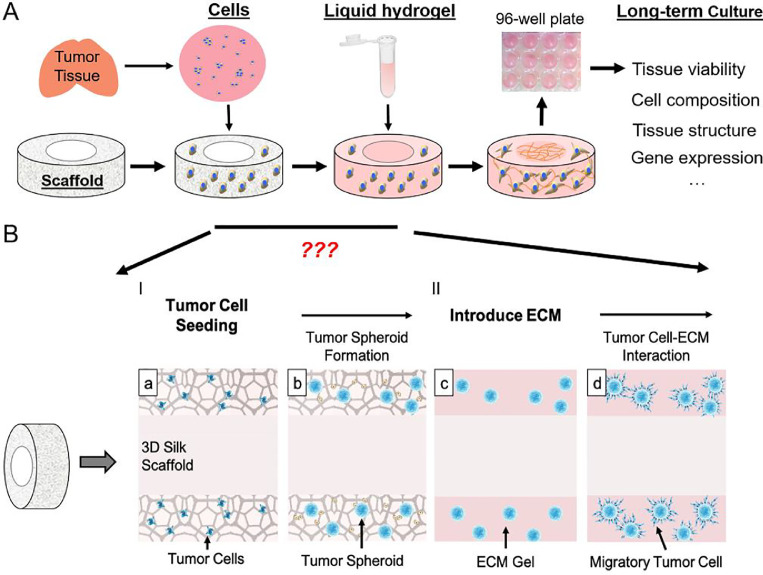
**Schematics of the 3D modeling process. A.** Schematics of the 3D brain tissue engineering process, as applied to rodent primary cortical neurons [[27], [28], [29],[33], [34]], neural stem cells [30] and human patients [32,35], with the methodology detailed in [31]. To adapt the process for brain tumor model, questions regarding media conditions, ECM and timing for the change of culture conditions need to be addressed, as illustrated in **B.I.** Dissociated tumor cells are seeded onto a donut-shaped 3D silk-based porous scaffold, from which tumor spheroid develops. **II.** ECM gels are introduced to the scaffold filling the pores and the center-hole (CH) region, providing a permissive environment for the migrating tumor cells and cell-cell interaction.

Tumor *in vivo* growth involves single cancer stem/initiating- cell residence, clonal expansion, ECM remodeling, and interactions with the microvasculature. To simulate the *in vivo* process, we built upon a 3D silk scaffold that we previously used for a bioengineered brain tissue model [[27], [28], [29]] to incorporate different tumor microenvironmental factors, i.e., ECM and soluble factors, for an *in vitro* tumor model with *in vivo*-like tissue structure (Figure 1). The donut-shaped scaffold has a center hole (CH) region that permits the incorporation of different matrix and cells than those in the surrounding scaffold region.

Patient tumor tissue has high variability regarding tumor type, grade, and post-surgical damage; some cells may proliferate within the first week, while others may suffer great initial loss and only recover after a prolonged period in culture. To adjust for these differences, we designed a workflow to adapt to each tumor's growth need at different stages. At each stage, different variations of the engineering input were tested, and the growth outcome was measured with phenotypic assays. At Stage I of tumor cell seeding, three media conditions were tested, NeuralBasal/B27 supplemented with fibroblast growth factor (FGF, 20 ng/mL) and epidermal growth factor (EGF, 20 ng/mL) as used for neural stem cells (“NB”), NeuralBasal/B27/EGF/FGF supplemented with 10% FBS (“N+FBS”), and “NB” media mixed at 1:1 ratio with VEGF-containing EGM-2 MV without serum (“NE”). At Stage II of ECM introduction, collagen gel alone or with Matrigel mix (at 1:1 by weight) was tested. These models were maintained in culture for up to 3 months and evaluated for tissue viability, morphology, and cell compositions. .

This study summarized the findings of our 3D brain tumor models from 11 pediatric tumor cases (Table I), consisting of three medulloblastoma (MB) patients, three ependymoma (EPN) patients, one glioblastoma (GBM) patient, and four juvenile pilocytic astrocytoma (Ast) patients.MB-1 and MB-2 are classified as group 3 and MB-3 as group 4 MB. All three EPN cases are posterior fossa tumor. In our clinical practice, only the supratentorialependymomas are tested for RELA/YAP1 mutations. Since the molecular data does not change our management with posterior fossa ependymomas at present, no test for molecular classification was done on the EPN cases in this study.

**Table 1:** Pediatric Brain Tumor Cases for Primary Culture.

**Type**	**Case ID**	**Grade**	**Age**	**Gender**	**Location & Molecular Classification**
Medulloblastoma (MB)	MB-1	IV	11 yr	M	Anaplastic/large cell; CTNNB1 negative; N-MYC non-amplified. Group 3
	MB-2	IV	5 yrs	M	Anaplastic; CTNNB1 negative; p53 non-mutated. Group 3
	MB-3	IV	8 yrs	M	Classic; CTNNB1 negative; MYC and N-MYC non-amplified. Group 4
Ependymoma (EPN)	EPN-1	II	15 months	M	Posterior fossa. no data on whether PFA or PFB
	EPN-2	II	11 months	M	Posterior fossa. no data on whether PFA or PFB
	EPN-3	III	2 yrs	F	Posterior fossa. no data on whether PFA or PFB
Glioblastoma (GBM)	GBM-1	IV	8 yrs	M	IDH1 negative; p53 non-mutated; MGMT non-methylated
Pilocytic astrocytoma (Ast)	Ast-1	I	16 yrs	M	Parietal. BRAF V600E
	Ast-2	I	14 yrs	F	Posterior fossa. Non BRAF mutated
	Ast-3	I	17 yrs	M	Frontal. Non BRAF mutated
	Ast-4	I	3 yrs	F	Cerebellum, KIAA1549/BRAF rearrangement

### Effects of culture media and ECM on 3D model viability

**Figure 2:**
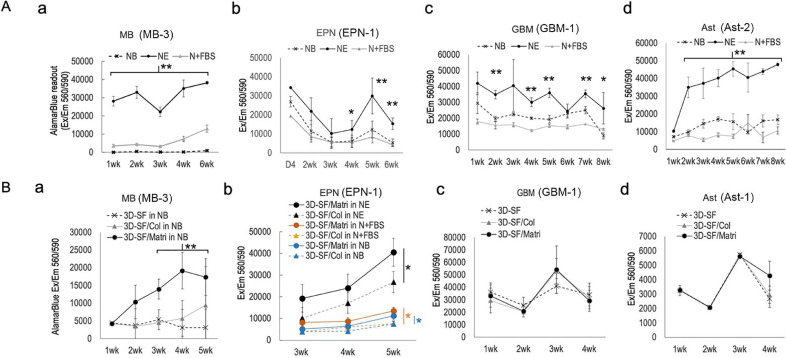
**3D brain tumor model viability assessment by AlamarBlue assay. A.** Effects of three conditions (e.g., NB, NE and N+FBS) on (**a**) medulloblastoma (MB), (**b**) ependymoma (EPN), (**c**) glioblastoma (GBM) and (**d**) pilocytic astrocytoma (Ast). Each chart is from a single tumor case as marked in each chart title..Four replicates were used for each data point per condition per time point. Error bar, standard error of mean. Student's t-test, *, p <0.05; **, p < 0.01. **B.** Effects of ECM gel types infused in 3D scaffolds, e.g., collagen type I (3D-SF/Col) or Matrigel (3D-SF/Matri), compared to 3D scaffold only (3D-SF) cultures. (**a-MB**), 3D cultures in NB media are shown here, demonstrating significant growth-promoting effects by Matrigel. This effect was not significant in cultures in NE media. (**b-EPN**), 3D cultures in all media and matrix combination conditions are compared, showing the growth-promoting effect of Matrigel in all three media conditions. (**c-GBM**) and (**d-Ast**) show 3D cultures in NE media. Each chart is from a single tumor case as marked in each chart title. Four replicates were used for each data point per condition per time point. Error bar, standard error of mean. Student's t-test: *, p <0.05; **, p < 0.01.

To evaluate 3D model viability, alamarBlue assay was used as we previously described for 3D brain tissue models [[27], [28], [29]] (Figure 2). Figure 2A shows the time courses of the assay readouts of 3D silk scaffold-only (3D-SF) models of representative tumor cases, a-MB, b-EPN, c-GBM, and d-Ast. The results show that NE media supported higher tissue viability throughout the culture duration than NB or N+FBS media for all tumor cases of different types we tested.

When comparing the role of ECM for 3D model growth, different tumor types showed different preferences for gel presence or types (Figure 2B). For MB models in NB media (Fig 2B-a), Matrigel (3D-SF/Matri) addition significantly improved model growth than SF-only models (3D-SF) or collagen gel addition (3D-SF/Col). For EPN models (Fig. 2B-b), Matrigel improved model viability compared to collagen gel in all three media conditions; and the combination of NE media and Matrigel resulted in the highest model viability. For models of glioma either high-grade GBM (Fig. 2B-c) or low-grade Ast (Fig. 2B-d), no significant differences were found between ECM gel types or between gel-infused and SF-only models.

### Physical substrate-dependent tumor growth

To examine how a physical substrate affects tumor growth, we compared tumor cells growing in 3D silk scaffolds alone versus with ECM gel infusion. We observed that the spheroid-forming ability correlated with tumor grade with the higher-grade types easier to develop into tumor spheroids, and we have had no success in forming tumor spheroids with low-grade gliomas (e.g., pilocytic astrocytoma). As an example,primary MB cultures (e.g. MB-1) in suspension immediately developed multicellular aggregates (Fig. S3-a). These self-assembled aggregates formed with great variability in size between batches and among tumor cases, thus unsuitable for down-stream applications such as drug screening.

To test what substrate is suitable for controlled tumor cell adhesion, we examined 2D surfaces in pilot studies. On a 2D surface with poly-lysine coating, MB cells developed neural-like processes (Fig. S3-b) of mixed neural lineages, e.g., with neuronal-like TUJ1+ or glial-like GFAP+ staining (Fig. S3-e). On 2D surfaces with diluted gel coating such as collagen (Fig. S3-c) or Matrigel (Fig. S3-d), cell aggregates formed shortly after 24hr, likely due to increased cell mobility rather than clonal expansion. With other tumor types, we also found that poly-lysine coating resulted most reliable tumor cell adhesion compared to other options; therefore, we chose poly-lysine coated 3D silk scaffolds for all subsequent 3D models.

**Figure 3:**
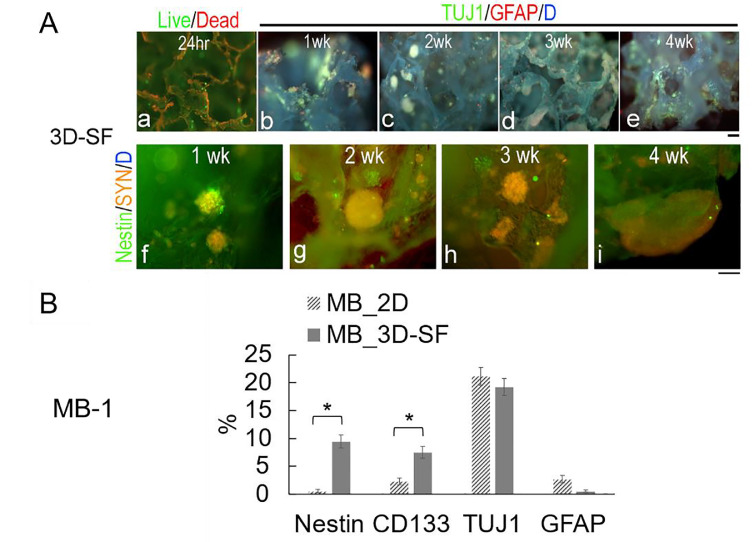
**Medulloblastoma 3D scaffold-only (3D-SF) cultures.** All data here are from MB-1.**A**. Representative immunofluorescence images of 3D scaffold-only (3D-SF) models. (**a**), A representative Live/Dead-stained image of a 3D model at 24 hr post cell-seeding, showing randomly distributed single live cells (*green*) throughout the scaffold that auto-fluoresced in red. (**b-e**), Representative 3D models triple-stained for TUJ1 (*green*), GFAP (*red*) and nuclei DAPI (*blue*), showing tumor spheroids (*greenish*) anchored within the pores of the scaffold grew in size over time, e.g, **b**-1wk, **c**-2wk, **d**-3wk, **e**-4wk. Note that the silk material autofluoresced in *blue*. Scale bar, 100 μm. **B**. Flow cytometry-measured cell percentages from MB 2D vs. 3D cultures of 3wks in NE media. Stem cell markers, Nestin and CD133 showed significantly higher cell proportions in 3D than 2D cultures, whereas differentiated cells such as TUJ1+ neuronal-like or GFAP+ glial-like cells showed no difference.

In a 3D silk scaffold, the initial cell seeding resulted in a random distribution of individual cells (Fig. 3A-a). Overtime, tumor spheroids developed from which neural-like processes were found to extend and course along the scaffold surface (Fig. 3A-b, c, d, e). Nestin+ (neural stem cell marker) cells were frequently found in tumor spheroids but not as single cells (Fig. 3A-f, g, h, i), suggesting that the tumor spheroids are derived from tumor stem/progenitor cells among the initial dissociated cell mixture.

To examine how the physical substrate can provide a selective growth advantage for certain cell types, we measured cell percentages of major neural cell markers (Fig. 3B). Tumor spheroids in 3D models contained significantly more Nestin+ (9.4 ± 1.2%) and CD133+ (putative cancer stem cell [18,38], 7.4 ± 1.1%) cells than 2D cultures (0.6 ± 0.4% and 2.4 ± 0.7%, respectively). In contrast, the more differentiated cells such TUJ1+ and GFAP+ cells showed no differences in their percentages on 3D-SFs versus 2D surfaces. These data suggest that the 3D physical environment is necessary to support stem/progenitor-like cells of tumor tissue.

### Gel-alone compared to 3D scaffold/gel composite models

**Figure 4:**
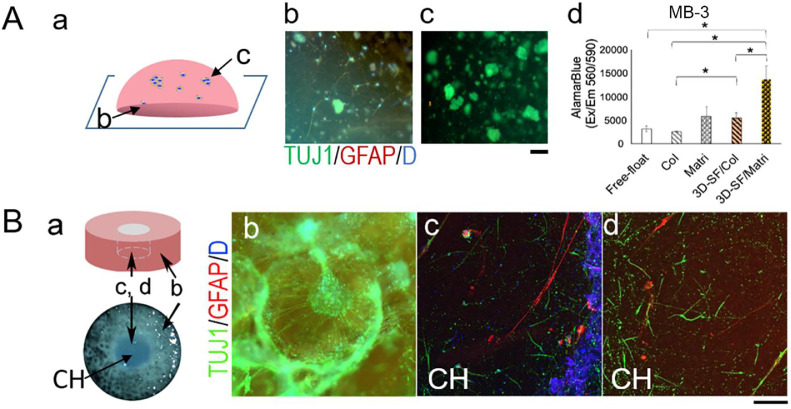
**Medulloblastoma gel-only cultures compared to 3D scaffold/gel composite models.** All data here are from MB-3.**A.** Gel-only cultures triple-stained for TUJ1 (*green*), GFAP (*red*) and nuclei DAPI (*blue*). Spheroids were embedded in gels (**a**-schematics), showing cells at the bottom spreading on the supporting dish surface (**b**) and cells in the interior of the gel (**c**). Scale bar, 100 um. (**d**), AlamarBlue assay comparing different 3D formats with the same cell source and seeding number, e.g., free-floating in suspension culture, embedded in collagen (Col) or Matrigel (Matri), in 3D scaffold infused with collagen (3D-SF/Col) or with Matrigel (3D-SF/Matri). Student's t-test: *, p <0.05. **B.** 3D scaffold/Matrigel composite model (**a**-schematics). Representative immunofluorescence images of 3D models triple-stained for TUJ1 (*green*), GFAP (red) and nuclei DAPI (*blue*). Tumor spheroids in the scaffold pore (**b**) were found to extend neuonal-like processes in the gel matrix, whereas tumor cells migrated into the gel-filled center hole (CH) region exhibited either glial-like phenotype (**c**, *red*) or neuron-like phenotype (**d**, *green*). Scale bar, 100 um.

Gel-embedded tumor cell cultures are the most common 3D models, therefore we compared gel-only and 3D scaffold-based cultures in pilot studies. We found considerable challenges adapting gel-only cultures to primary brain tumor cells, for example, there was drastic initial cell death resulting in dead cells entrapped in gels and gel instability in long-term cultures. Fig. 4A shows an example of primary MB (e.g. MB-3) tumor spheroids embedded in collagen and Matrigel mixture after 4 weeks of culture. Notably, cells in contact with the well bottom (Fig. 4A-b) were found to extend processes on the stiffer surface whereas those in the interior of the gel (Fig. 4A-c) had little morphological change. AlamarBlue assay results show that the presence of 3D scaffold significantly improved the viability of 3D-SF/gel composite models than gel-alone cultures (Fig. 4A-d).

Fig. 4B shows an example of a Matrigel-infused MB model, demonstrating significant tissue remodeling in 3D-SF supported gels. In particular, the scaffold provided structural support for long TUJ1+ processes extending from the spheroids and into the gel matrix (Fig. 4B-b). In the gel-only CH region (see Fig. 4B-a schematics), both GFAP+ (Fig. 4B-c) and TUJ1+ (Fig. 4B-d) cells were found infiltrating into the matrix. Together, these data suggest that the 3D scaffold and hydrogel can offer different structural support for tumor cell adhesion, spreading, and motility for 3D growth, and that there are synergistic benefits when both are combined in a 3D model.

### Tumor type-specific 3D growth pattern

**Figure 5:**
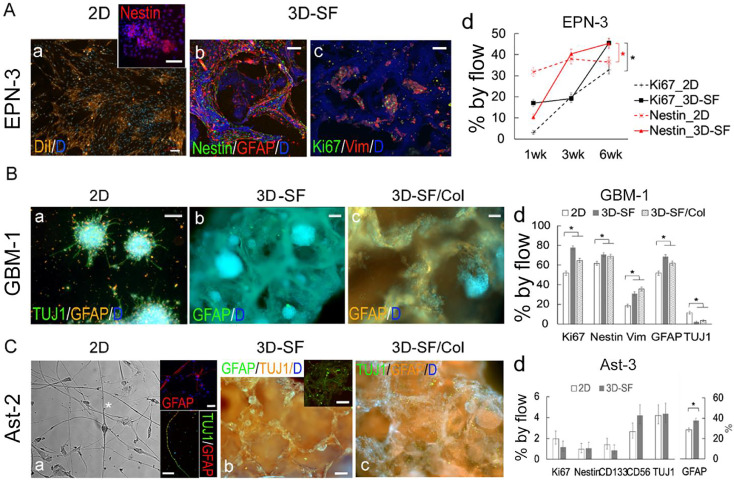
3D **cultures of ependymoma, glioblastoma and pilocytic astrocytoma.** The corresponding tumor case for each piece of data is specified.**A.** Ependymoma. (**a**), 2D cultures co-stained for DiI (*red*) and DAPI (*blue*). The clusters of higher cell density were found to be enriched with Nestin+ (*red*) cells (*inset*). (**b**), 3D cultures triple-stained for Nestin (*green*), GFAP (*red*) and nuclei DAPI (*blue*). (**c**), 3D cultures triple-stained for mitotic marker Ki67 (*green*), GFAP (*red*) and nuclei DAPI (*blue*). Scale bar, 100 um. (**d**), Flow cytometry-measured cell percentages showing time-dependent increases of Ki67+ and Nestin+ cell proportions in both 2D and 3D cultures in NE media. At 6wk, 3D-SF models showed significantly more Ki67+ and Nestin+ cells than 2D cultures. Error bar, calculated confidence intervals (see [32]). **B. Glioblastoma**. (**a**), 2D cultures triple-stained for TUJ1 (*green*), GFAP (*red*) and nuclei DAPI (*blue*), showing prominent tumor spheroids. (**b-c**), 3D cultures co-stained for GFAP (*red*) and DAPI (*blue*) in 3D-SF models (**b**) and scaffold infused with collagen gel (**c**). (**d**), Flow cytometry-measured cell percentages, comparing 2D vs. 3D-SF and 3D-SF/Col cultures of 3wks in NE media, all from a single tumor case. Error bar, statistical construction of simultaneous confidence intervals; *, p <0.05 (see [32]). **C. Pilocytic astrocytoma**. (**a**), 2D cultures in phase-contrast light imaging, showing a mixture of cell morphologies, including bi-polar shaped cells (asterisk) that we previously observed in normal brain cell primary cultures as astro-neuronal cells [32]. The long processes were either stained with GFAP (*red*) or co-stained with both TUJ1 (*green*) and GFAP (*red*) (*inset*).). (**b-c**), 3D cultures triple-stained for TUJ1 (*red* in **b** and *green* in **c**), GFAP (*green* in **b** and *red* in **c**) and nuclei DAPI (*blue*) in 3D-SF models (**b**) and scaffold infused with collagen gel (**c**). (**d**), Flow cytometry-measured cell percentages, comparing 2D vs. 3D-SF cultures of 4wks in NE media, both from a single tumor case. Error bar, statistical construction of simultaneous confidence intervals; *, p <0.05 (see [32]).

In addition to medulloblastoma, we compared other major pediatric brain tumor types, including ependymoma, glioblastoma, and pilocytic astrocytoma regarding their 3D model growth pattern (Figure 5). 2D companion cultures were used as controls. For EPN, DiI staining showed the tumor cells tended to grow as a monolayer with loci of high cell density (Fig. 5A-a). These loci were found to be enriched with Nestin+ cells (*inset*). In 3D-SFs, tumor cells enveloped the scaffold surface consisting of mostly GFAP+ cells and some Nestin+ cells (Fig. 5A-b). After significant surface coverage at 3-6 weeks, cell aggregates emerged throughout the scaffold with a preference to tight corners of the pores (Fig. 5A-c). The enrichment of Ki67+ cells in these aggregates suggests that these multicellular structures constitute the growth niche but not the monolayer-forming cells. Flow analysis showed that both Ki67+ and Nestin+ cells had time-dependent increases in culture (Fig. 5A-d). Together, these data suggest that EPN growth in 3D consists of growth niches of stem/progenitor-like cells and monolayer-forming cells.

For GBM, tumor spheroids quickly developed from single-cell clones even in 2D cultures (Fig. 5B-a). In 3D-SFs (Fig. 5B-b), abundant spheroids were found throughout the scaffold. In 3D-SF/ECM gel models (Fig. 5B-c), cell migration onto the scaffold surface and into the matrix was rapid and rampant. Flow analysis (Fig. 5B-d) showed that both 3D-SF and 3D-SF/Col models had higher percentages of Ki67+ replicating cells, stem-like Nestin+ cells, and Vim+ cells, and GFAP+ cells compared to 2D cultures, but negligible CD133+ or TUJ1+ cells (<2%). Together with the viability assay results shown in Fig. 2, these data suggest that high-grade gliomas such as GBM have robust *in vitro* growth that is not as sensitive to ECM gel types as other tumor types such as MB or EPN.

For pilocytic astrocytoma, despite the improved growth in NE media (see Fig 2), there were challenges in keeping most of the dissociated cells alive initially (similar to our experience with normal brain tissue [32]) and expanding the slow-growing surviving cells into sufficient numbers. Mixed types were observed in 2D cultures (Fig. 5C), such as bipolar-shaped with a long process, neural-like with branched processes and flat fibroblast-like cells. The branched processes were stained positive for GFAP, whereas the long processes co-stained for both TUJ1 and GFAP (inset), similar to the dual identity astro-neuronal cells we observed with primary cultures of normal brain tissue [32]. The surviving cells can remain stable for months with little change in morphology or number. In 3D-SFs, tumor spheroid formation varied considerably depending on individual tumor cases, from sparse (i.e., 1-3 spheroids per scaffold) to relatively abundant (i.e., >10 spheroids per scaffold, Fig. 5C-b). Notably, all Ast cases in this study showed negligible proportions of Nestin+ or CD133+ cells or Ki67+ cells (<2%), as shown in Fig. 4C-d for Ast-3.

### Transcriptomic profiles of 3D tumor models compared to the primary tumor tissue

To compare gene-level differences of the *in vitro* models from their primary tumor tissue source, we used RNAseq. We tested for differential expression between cultures and primary tissue for each tumor type using edgeR. The number of differentially expressed genes (adj. p < 0.05) identified in each of the comparisons between *in vitro vs.* primary tissues, are 104 (0.6%) of medulloblastoma, 3,392 (20.2%) of ependymoma, and 576 (3.4%) of astrocytoma, out of total 16,795 protein-coding genes and lincRNAs.

**Figure 6:**
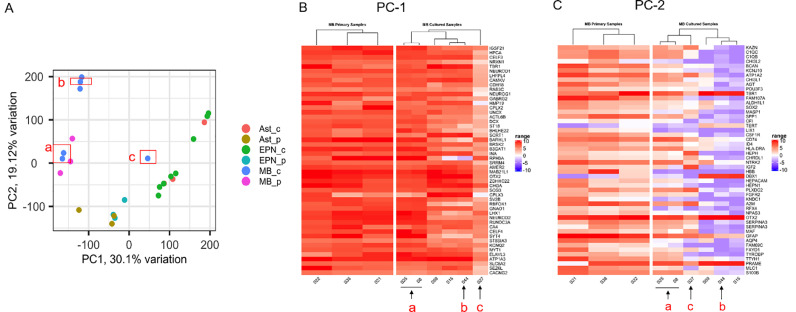
**Transcriptomic analysis of 3D tumor models (_c) in comparison with the original tumor (_p) tissue.** All cultures used late time-points (6-12 wks) for this analysis. **A.** Principal component analysis (PCA) plot. Total sequenced genes in the top 90% ranked by variance were used for the plot. Red boxes highlight the 3D model versions all derived from MB-3: **a,** 3D-SF only in NE media and differed by time points (6wk versus 10wk); **b,** 3D-SF/Matri in NE media at 6wk; **c,** 3D-SF in N+FBS media at 6wk. **B.** Heatmap of log-transformed gene counts of the top 50 genes responsible for PC1 variance, comparing medulloblastoma primary tumor and their 3D models. **C.** Heatmap of log-transformed gene counts of the top 50 genes responsible for PC2 variance, comparing medulloblastoma primary tumor and their 3D models. (**a, b, c**) in **B & C** correspond to those marked in **A**.

Figure 6 shows the principal component analysis (PCA) plot of the samples from three tumor types, MB (3 primary, 6 cultures), EPN (3 primary, 8 cultures) and Ast (3 primary, 3 cultures; the one GBM case is not included). Due to resource constraints, we did not have duplicates for this analysis: each model is unique and models from the same patient vary by model conditions. The result demonstrates that the 3D MB models (MB_c) overlap with the primary tumors (MB_p) in either principal component PC1 or PC2. In addition, two models derived from the same patient (MB-3) clustered together with the patient-matched primary tumor tissue (Fig.6A-a); both models were 3D-SF only in NE media and differed by a 1-mo gap in culture (i.e., 6wk versus 10wk). The one model separated by PC1 was 3D-SF in N+FBS media (Fig.6A-c). The other models separated by PC2 were 3D-SF/ECM gel models (Fig.6A-b). The clustering of MB models with the primary tumor tissue provides evidence that our 3D MB models preserved the gene expression patterns of the primary MB tissue.

To understand how the different *in vitro* conditions affect the gene signatures of the tumor cells compared to their *in vivo* source, we plotted the top 50 genes ranked by variance for the respective principal components of PC1 (Fig. 6B) or PC2 (Fig. 6C). Interestingly, the genes responsible for PC1 variance are either associated with neuronal development (NEUROG1, CPLX2, UNCX, etc) or neuronal function including synaptic transmission and ion channels (HPCA, SV2B, SYT4, KCNQ2, CACNG2, etc). The genes responsible for PC2 variance include extracellular matrix remodeling (BCAN, CHI3L1, CHI3L2), complement activation (C1QC, C1QB, CF1), cytoskeletal regulation (HPACAM, CD74), growth factors (FGFR2, IGF2, CSF1R), suggesting cell-ECM interaction activated pathways.

However, our EPN and Ast models did not cluster with their primary tumors. EPN models, separated by a range of distances from the cluster of the three primary tumors. Our ongoing analysis suggests that a lack of additional supporting microenvironment may play a role in the discrepancy between *in vitro* models and the native tissue. For Ast, we did not find noticeable pattern of clustering despite various methods we attempted for the PCA plot. At present, our study did not have statistical power for further analysis, due to the small sample size and the inherent heterogeneity of juvenile pilocytic astrocytoma [51].

**Figure 7:**
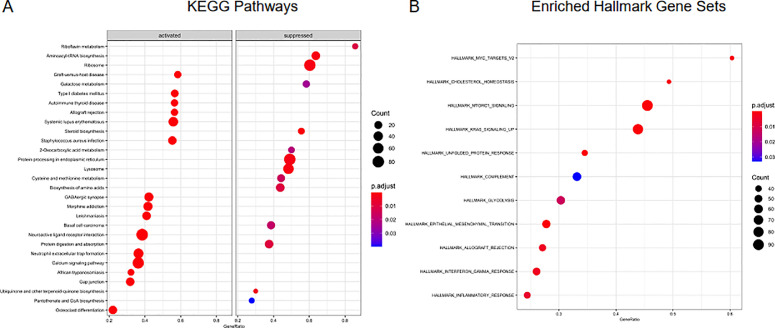
**Pathway analysis of medulloblastoma (MB) primary versus cultured samples. A.** GSEA (Gene Set Enrichment Analysis) performed using the entire filtered gene list n=16,795. Log transformed normalized counts (cpm- counts per million) were used to obtain enriched KEGG pathways with pvalue< 0.05. **B.** Enriched pathways using “hallmark” gene sets as part of MSigDB with pvalue< 0.05.

To determine what pathways were involved in the differences between the primary tumor and the cultured samples, GSEA (Gene Set Enrichment Analysis) was performed using the entire filtered gene list n=16,795. Fig. 7 shows the pathway analysis results for MB, as enriched KEGG pathways of activation or suppression by the DEGs between the primary and cultured samples (Fig. 7**A**), or as enriched pathways using the “hallmark” gene sets as part of MSigDB (Fig. 7**B**). Additional pathway analysis for EPN and Ast are reported in Supplementary Figures **S1** and **S2** respectively.

## Discussion

Not all brain tumors can establish primary cultures *in vitro*. Lack of the brain microenvironment hampers the effort in recapitulating the primary tissue with 2D cultures or 3D spheroid/organoid systems. In this study, we aimed to reconstitute patient brain tumor cells into 3D tumor tissue for a range of brain tumor types. We based our studies on a bioengineered normal brain cortical tissue model that we have established using a 3D silk fibroin-based porous scaffold as the structural base [[27], [28], [29]]. As a first step, this report defined the starting conditions with regards to chemically defined media, ECM components, and 3D formats (e.g., suspension, in 3D-SF only or 3D-SF/gel composite). These comparisons show that the 3D scaffold structure plays an important role in supporting tumor spheroids, providing structural stability to gels and preserving tumor stem cells in 3D. We determined the culture conditions that yielded the most robust *in vitro* growth of the patient's primary tumor cells in 3D. The different phenotypes of these models revealed tumor type-specific biology, including different tumor stem cell niches and cell type changes to their microenvironment. Finally, a comparison of the transcriptomic profiles between patient's tumor tissue and patient-derived tumor models allowed us to determine the tumor models best representative of the primary tumor's gene signatures. In particular, the 3D MB model in a scaffold-only format in NE media exhibited nearly indistinguishable transcriptomic profiles from the patient-matched tumor with <1% gene expression differences. In addition, pilocytic astrocytoma, the most common low-grade brain tumor type that has no established cell lines and is known to be difficult to culture *in vitro*, was supported by our defined media and the 3D models. Detailed analysis of the gene signature differences between the primary tissue and the *in vitro* models provided insights for future model optimization efforts.

Considering a tumor's heterogeneous cell composition, the starting culture media would ideally be pro-growth of all cell types of the primary tumor tissue; however, there is a lack of understanding of growth factors in the microenvironment of different brain tumors. The conventional approach is to supplement serum to a base medium especially for difficult-to-culture tissue types. However, the serum is not naturally encountered by the brain tissue, and we had previously observed detrimental effects of serum on primary cultures of normal human brain cells [32]. Similarly, in this study, we found that serum presence inhibited the growth of primary brain tumor cells, one of the effects being suppressing cancer stem-like cell growth. We identified the combined medium of pro-neural Neurobasal/B27 and pro-endothelial EGM media as pro-growth for all major brain tumor types (e.g., MB, EPN, GBM, and Ast). Since we did not observe endogenous endothelial cells in the tumor models, the improved growth may be attributed to VEGF given its important role in neurovascular interactions in the brain [39], [40], [41]. Our ongoing studies continue to define tumor type-specific growth factors for culture media optimization by examining tumor secretomic profiles. Nevertheless, the starting culture condition as identified in this study that supports a wide range of primary tumor types is a significant step forward by providing the vital cell source that is currently unavailable for studies.

Our model is designed to combine the need for ECM-independent clonal expansion of tumor spheroid and ECM-mediated 3D cell-cell interaction. Our data showed that compared to spheroid-only suspension cultures, the 3D silk scaffold can better capture tumor cells and support tumor types of different spheroid-forming abilities. Studies of spheroid cultures have shown that the spheroid-forming ability varies considerably from one tumor to another, in part due to the differences in cancer stem cell proportions in each sample [42]. Additionally, non-spheroid forming cells may be needed for 3D spheroid growth. For example, in the EPN models, it is found that the spheroid-like growth niche requires other monolayer-forming cells that cannot be adequately captured in the suspension culture. The role of the 3D silk scaffold in supporting tumor spheroid growth can be attributed to the 3D structure and the brain-mimetic stiffness. The pores can not only increase the surface areas for single-cell adhesion but also provide the corners for spheroid anchoring. Other studies have found that the substrate stiffness can modulate GBM cell fate by activating matrix-degrading enzymes for ECM deposition and remodeling and RhoA/Ras/ROCK genes for mechano-sensing pathways [[43], [50]]. With regards to the silk material in our model, the stiffness of its surface falls into the range of the Young's modulus that supports neural cell growth [44], and the bulk property of the composite structure is similar to that of the cortical tissue [29]. Surprisingly, the 3D-SF only model is sufficient to preserve the gene signatures of medulloblastoma in one of the two MB patients with RNAseq profiles. The ability of the 3D model to accommodate tumor types of different spheroid-forming abilities would expand its appeal as a model platform for brain tumors.

Our studies used a unified model system to demonstrate key differences of tumor cell sensitivity to its microenvironment depending on tumor type. For example, MB and EPN preferred Matrigel but GBM and Ast showed indifference compared to 3D-SF only models. Matrix remodeling by tumor cells has been extensively studied in gel-alone systems using a wide range of biomaterials, including poly(ethylene-glycol) (PEG), hyaluronic acid, gelatin and collagen, etc [43,[45], [46], [47], [48], [49]]. These studies have shown that proteolytic degradation of the matrix by the tumor cells is necessary for structural remodeling, and that different ECM matrices can activate different signaling pathways in the cells. Such a reciprocal relationship between brain tumor cells and its ECM environment is demonstrated by a previous study using a similar silk scaffold model system showing different MMP release profiles of GBM and EPN and how these activities were differentially activated in different ECM contexts [35]. In this study, we expanded the analysis to other brain tumor types and include a comparison with the original tumor tissue. Our data showed that cell-ECM interaction can activate functional pathways in tumor cells: for example, the genes responsible for PC1 variance in the PCA plot that separate 3D-SF/gel model from the primary MB tissue are either associated with neuronal development (NEUROG1, CPLX2, UNCX, etc) or neuronal function including synaptic transmission and ion channels (HPCA, SV2B, SYT4, KCNQ2, CACNG2, etc). While the implication for tumor growth is unclear, these insights present intriguing questions linking normal neuronal function to medulloblastoma.

The novel finding of this study is the match of transcriptomic profiles of *in vitro* models with that of the patient-matched primary tumor, based on one medulloblastoma case. To our knowledge, this is the first report showing the transcriptomic profiles of *in vitro* models matching that of a patient's original tumor. The flaw of n=1 for this conclusion combined with the small sample sizes of each tumor type limit generalization of the study's transcriptomic findings. In contrast, our EPN and Ast models did not cluster with their primary tumors. EPN models exhibited a range of distances separate from the cluster of the primary tumors. Our ongoing analysis suggests that additional extracellular microenvironment may be needed to further improve the degree of biomimicry of EPN *in vitro* models to the *in vivo* tumor. For Ast, our study did not have the statistical power for detailed analysis, due to the small sample size and the inherent heterogeneity of juvenile pilocytic astrocytoma [51]. The lack of clustering of the four pilocytic astrocytoma samples is consistent with their highly variable phenotypic expressions such as cell type compositions and spheroid-forming abilities observed in our study, suggesting large-scale studies are warranted for subtype classification. Additionally, recent studies suggest a critical role of normal neuronal activity in glioma growth and progression [52]. Modified 3D model design to incorporate a normal brain compartment can be beneficial to model these difficult-to-culture brain tumor types. Accordingly, deciphering and differentiating the respective contributions of normal neural cells versus transformed tumor cells will need deconvolution of cell-type-specific gene expression profiles by digital cytometry [53] or single cell RNA-sequencing.

In this study, a biopsy of as little as 100-milligram of tissue can generate hundreds of these 3D models that can fit into 96-well plates for downstream drug studies. Once validated to be representative of the original tumor tissue, these models can potentially be used for concurrent drug testing with the patient's ongoing chemotherapy. A notable challenge using primary human tissue is the availability of tumor cases; for example, MB affects 400 patients a year in the US and only a couple of cases on average a year in our hospital, limiting our efforts in replicating our findings in a timely manner. Thus, future modeling efforts for MB need to devote to expanding and renewing the primary cell/model source, such as from cryopreserved samples or using serial passage, while preserving primary tissue characteristics. On the other hand, further optimization of the 3D culture conditions is needed to improve the initial cell numbers for low-grade gliomas such as pilocytic astrocytoma. We envisioned that using the 3D scaffold-based bioengineered tumor models in brain cancer research will soon be established as an important component of bench-scale platforms for use in both *in vitro* and *in vivo* settings.

## Conclusions

Lack of the brain microenvironment hampers the effort in recapitulating the primary tissue with 2D cultures or 3D spheroid/organoid systems. In this study, we aimed to reconstitute patient brain tumor cells into 3D tumor tissue based on an established, bioengineered normal brain cortical tissue model using a 3D silk fibroin-based porous scaffold as the structural base [[27], [28], [29], [30], [31], [32], [33], [34], [35]]. This report showed that the 3D scaffold base structure plays an important role in supporting tumor spheroids, providing structural stability to gels, and preserving tumor stem cells in 3D. The 3D tumor models exhibited tumor type-specific biology, including different tumor stem cell niches and cell type changes to their microenvironment. Finally, we found that in one out of two cases of medulloblastoma with RNAseq data, the 3D models in a scaffold-only format in combined pro-neural and pro-endothelial cell growth media can preserve nearly indistinguishable transcriptomic signatures from the patient-matched tumor; while all three cases in this study collectively had 0.6% differences in gene expression between *in vitro* models and primary medulloblastoma tissue. In addition, juvenile pilocytic astrocytoma, the most common low-grade brain tumor type that has no established cell lines and is known to be difficult to culture *in vitro*, was supported by our defined media and the 3D models. Insights from the comparison of the gene signature differences between the primary tissue and their *in vitro* models will facilitate future model optimization efforts.

## Author Contributions

M.D. T-S. conceived and designed the study, performed the experiments, collected and analyzed the data, prepared the figures including the schematics drawing, and wrote the manuscript. W.W. assisted with statistical analysis. H.C. and J.G. provided transcriptomic analysis. M.J.B. provided human patient tissue. C.L.L provided clinical input. All authors read and approved the final manuscript.

## Funding

We acknowledge support from Connecticut Children's Medical Center (CCMC) Strategic Research Fund (to M.D. T-S.), UConn Health Stimulus Award (to M.D. T-S) and CCMC Surgical Research Seed Grant (to M.D. T-S.), the Martin J. Gavin Endowment Fund (to CCL), and the National Institutes of Health (CA195712; P30 JAX Cancer Center grant). Part of the study was funded by the American Brain Tumor Association Discovery Grant from an Anonymous Family Foundation to M.D. T-S (DG1900023). M.D. T-S first started the study in UConn Health, continued after she moved to JAX and finished the study at JAX.

## Institutional review board statement

Not Applicable.

## Informed consent statement

Not Applicable

## Data availability statement

TheRNAseq data presented in this study can be accessed from the Sequence Read Archive (SRA) on NCBI website (SUB10713222). Other raw data and details of the modelscan be provided upon request by contacting the Corresponding Author.The primary tumor tissues and *in vitro* models used in this study can be requested from Ching Lau (ching.lau@jax.org) at the Jackson Laboratory.

## Conflicts of Interest

The authors declare no conflict of interest. The funders had no role in the design of the study; in the collection, analyses, or interpretation of data; in the writing of the manuscript, or in the decision to publish the results.

## Appendix Supplementary materials

Image, application 1
